# Good Bye Lenin Revisited: East-West Preferences Three Decades after German Reunification

**DOI:** 10.1515/ger-2022-0042

**Published:** 2023-02-28

**Authors:** Mariia Bondar, Nicola Fuchs-Schündeln

**Affiliations:** Charles River Associates, Munich, Germany; Goethe University Frankfurt and CEPR, Frankfurt, Germany

**Keywords:** German reunification, preferences for redistribution, endogenous preferences

## Abstract

In this paper, we document that living under Communism versus Capitalism has lasting effects on preferences for a strong government. Relying on the natural experiment of German reunification and extending the analysis of Alesina and Fuchs-Schündeln (2007), we show that East Germans still have stronger preferences for redistribution than West Germans 27 years after reunification. While convergence of preferences occurs, the speed of convergence decreases significantly over time. Evidence from cohorts born after German reunification points towards significant intergenerational transmission of preferences.

## 1 Introduction

Experiences shape preferences. This has meanwhile been shown for a wide range of preferences, from risk and inflation tolerance (Malmendier and Nagel 2011; Malmendier 2021) to support of democracy (Fuchs-Schündeln and Schündeln 2015). Alesina and Fuchs-Schündeln (2007) analyze the effect of having lived under Communism versus Capitalism on preferences for a strong welfare state. Based on the 1997 and 2002 releases of the German Socio-Economic Panel, Alesina and Fuchs-Schündeln (2007) find East Germans to be significantly more in favor of the government taking a strong role in caring for vulnerable groups than West Germans. At the same time, they document convergence of preferences between 1997 and 2002 and conclude that it would take 20–40 years for the views of East and West Germans to converge if convergence were to continue at the same (linear) speed. By documenting convergence of preferences on the individual level, and stronger East-West differences for older individuals who lived under the different systems for a longer time, they can establish a causal effect of living under different systems on preferences.

In this paper, we revisit the long-term effects of having lived under Communism versus Capitalism on preferences. Adding the 2017 release of the German Socio-Economic Panel, we update the results obtained by Alesina and Fuchs-Schündeln (2007). We show that East Germans continue to be significantly more pro-state compared to West Germans even 27 years post reunification. Moreover, we find that the speed of convergence slowed down significantly. The convergence of preferences between 2002 and 2017 is mainly driven by the change in the composition of the population, with younger cohorts replacing older cohorts: On the individual level, there was on average no further significant convergence of preferences between 2002 and 2017. Finally, we present evidence on the intergenerational transmission of preferences: we find that young individuals born after reunification to parents who used to live in the East are significantly more likely to exhibit pro-state preferences than young individuals born to parents who used to live in the West, independent of their own current residence. Linking children to their parents, we document a strong correlation between the preferences of parents and children. This intergenerational transmission of preferences implies that the effects of political regimes on preferences can be extremely persistent.

This paper is closely related to a growing strand of literature on the formation of economic and political preferences. Ravallion and Lokshin (2000) and Corneo and Grüner (2002), among others, show that preferences for redistribution depend on income and perceived social mobility. Di Tella et al. (2007) find that land titles increase pro-market materialist attitudes. Guiso et al. (2006) and Luttmer and Singhal (2011) show that ethnicity and culture of the birth country are significantly correlated with preferences for redistribution among second-generation migrants. Alesina and Giuliano (2011) provide an overview of the theoretical literature and conclude that both personal (age, gender, ethnicity, socioeconomic status) and societal (political ideology, cultural norms) characteristics have an impact on preferences for redistribution. Fong (2001), Alesina and La Ferrara (2005), and Durante et al. (2014) establish a connection between preferences for redistribution and other values and beliefs such as concern for fairness, risk attitude, preferences regarding efficiency and equality, and belief in luck.

Following Alesina and Fuchs-Schündeln (2007), several studies have looked at political and social preferences in the context of German reunification. Svallfors (2010) uses the International Social Survey Program data for 1990, 1996, and 2006. He finds that attitudes towards state intervention of East Germans converge quickly to those of West Germans, and that there is no East-West difference in attitudes among the younger cohorts. Brosig-Koch et al. (2011), on the other hand, see no convergence in attitudes towards solidarity, cooperation, and fairness between East and West Germans, relying on laboratory experiments among students. Finally, Avdeenko (2018) shows that East Germans living in the border regions are less likely to vote for left parties, which the author interprets as retrospective electoral punishment.

An increasing number of studies use German reunification as a natural experiment in other contexts than the effect on preferences, see Fuchs-Schündeln and Hassan (2016) for an overview. If separation and reunification of Germany were exogenous, and there were no East-West differences prior to the separation in 1949, then West Germans constitute a valid control group for East Germans, and the differences in outcome variables right after reunification can be attributed to having lived under different economic and political regimes. The literature evaluates the effect of Communism on a wide range of variables, including savings (Fuchs-Schündeln 2008; Fuchs-Schündeln and Schündeln 2005), income and economic development (Burchardi and Hassan 2013), productivity (Burda and Severgnini 2018), labor market outcomes (Fuchs-Schündeln and Masella 2016), inflation expectations (Goldfayn-Frank and Wohlfart 2020), stock market participation (Fuchs-Schündeln and Haliassos 2021; Laudenbach et al. 2020), financial literacy (Davoli and Hou 2021) and gender norms (Bauernschuster and Rainer 2012; Lippmann et al. 2020). Becker et al. (2020), however, question the validity of German reunification as a natural experiment. They document pre-existing differences between East and West before separation and argue that there was selective out-migration from East Germany between 1949 and 1961. Following the approach taken by Alesina and Fuchs-Schündeln (2007), we argue that we are immune to this criticism. First, we show that the length of experiences with the different regimes is correlated with differences in preferences. Second, we analyze convergence not only on the aggregate, but also on the individual level.

The paper is organized as follows. Section 2 describes the data source and the sample. Section 3 replicates and expands the main analyses of Alesina and Fuchs-Schündeln (2007), including the new data wave from 2017. Section 4 analyzes the preferences of cohorts born after reunification. Finally, Section 5 concludes.

## 2 Data and Methodology

### 2.1 The German Socio-Economic Panel

As in Alesina and Fuchs-Schündeln (2007), we use the German Socio-Economic Panel (SOEP) as our primary data source. SOEP is a large representative panel of German households.1Socio-Economic Panel (SOEP), data for years 1984–2018, version 35, SOEP, 2019, doi:10.5684/soep.v35. The survey started in 1984, first sampling only West German households, but adding an East German sample already in the spring of 1990. The respondents of the survey answer a wide array of questions, providing information about their socio-economic background, family ties, labor market experiences, attitudes, and political views. The question concerning preferences about the role of the government in providing financial security was asked only in the 1997, 2002, and 2017 waves. This question reads: “In addition to the state, private individuals such as free-market companies, organizations, associations or individual citizens are responsible for a large number of social tasks in our society today. What is your opinion on who should be responsible for the following areas: financial protection of families/financial security for unemployment/financial protection in case of illness/financial security in old age/financial security for people in need of care”. The answers are given on a scale of 1–5, which correspond to “only the state”, “mostly the state”, “both the state and private forces”, “especially private forces”, and “only private forces”. We use the answers to this question to generate five independent dummy variables. Specifically, the dummy variables take the value of 1 when the respondent states that financial security is the responsibility of “only the state” or “mostly the state”, and the value of 0 otherwise. Thus, our dependent variables indicate whether an individual has pro-state preferences. Our main explanatory variable is a dummy variable *East*, which is equal to 1 if the respondent lived in East Germany before reunification, independent of the current residence.

Our basic sample is constructed as follows. For the main analyses, we use data from both individual and household questionnaires for 1997, 2002, and 2017. We only include individuals born before 1989. We use the initial West and East German samples (A and C), as well as all general refreshment samples (samples E, F, H, J, and K). Appendix Tables A1 and A2 provide summary statistics of the preferences separately for the East and West samples and years, once on the full sample and once on the balanced sample that includes only individuals who answer in all three survey waves. On average, East Germans in the unbalanced sample spent roughly 30 years under Communism. For the analysis of the youngest cohorts in Section 4, we focus on individuals born between 1990 and 1999, and link them to their parents based on the biography questionnaire.

**Figure 1:**
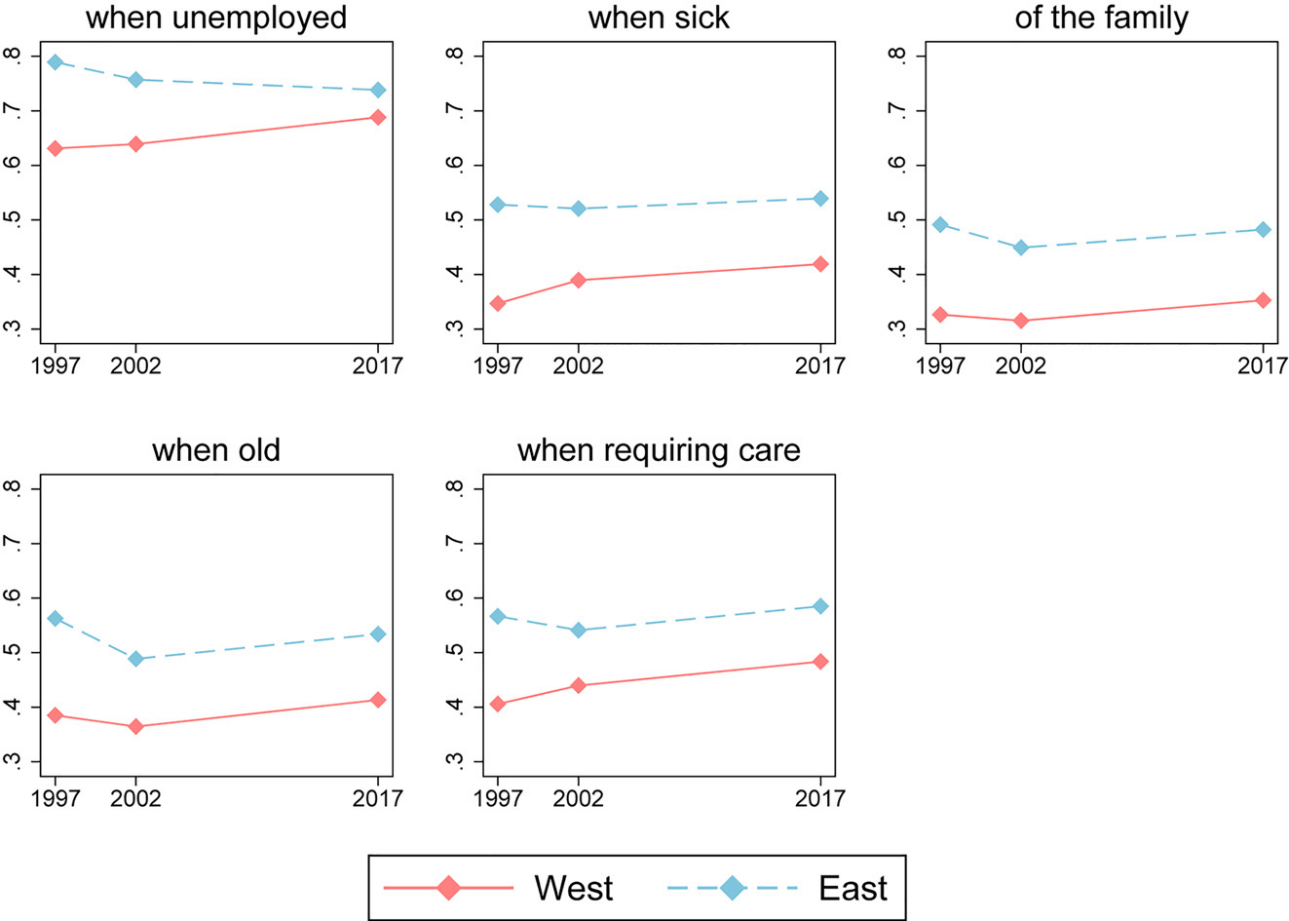
Fraction of the respondents from West and East who believe that the financial security is the responsibility of the state. Unbalanced sample.

Figure 1 shows the share of the respondents from West and East who state that the government should be responsible for providing financial security for the five different vulnerable groups. The majority of the respondents agree that the state should provide financial support to the unemployed – in 2017, 74% of East Germans and 69% of West Germans had pro-state preferences with respect to this question. At the same time, only 48% of the respondents from the East and 35% of the respondents from the West think that it is the government’s duty to support the family, and the averages are in a similar range for the other outcome variables. Over time, the West German sample becomes more pro-state: the fraction of individuals who consider that the state is responsible for financial security increases between 1997 and 2017 across all five variables. The East German sample, on the other hand, becomes on average less pro-state between 1997 and 2002. However, this pattern reverses between 2002 and 2017, when the opinion moves more pro-state in both samples, with the exception of the opinion on the state taking care of unemployed individuals in the East sample.

### 2.2 Estimation Methodology

Following Alesina and Fuchs-Schündeln (2007), we run probit regressions with robust standard errors clustered at the individual level to estimate the effect of living under Communism on preferences about the role of the state, as depicted in Equation (1).2
Appendix Tables A14 and A15 replicate the main results using linear probability model. The results remain unchanged.

(1) $$\mathrm{Pr}\left(Y=1|X\right)=\Phi \left(East+Year\;\;2002+{East}^{*}2002+Year\;\;2017+{East}^{*}2017+X\right)$$

In addition to the main explanatory variable *East* – which refers to the residence before reunification, independent of the current residence – we include two year dummies, as well as interaction terms between *East* and the year dummies (East*2002 and East*2017) to evaluate convergence of preferences between East and West Germans. The vector of control variables **X** includes age, its square and cube, gender, number of children and number of adults in the household, dummy variables for education (with fewer than nine years of schooling as the omitted category), dummy variables for marital status (with single as the omitted category), dummy variables for labor force status (with employed as the omitted category), dummy variables for the type of work (with blue-collar as the omitted category),3These variables take a value of zero for those who are not employed. and the log of household income. Explanatory variables are summarized in Appendix Tables A3 for the unbalanced sample and A4 for the balanced sample.

## 3 Main Results

Table 1 replicates the basic regression specification from Alesina and Fuchs-Schündeln (2007), but extends the sample to include data from 2017, in addition to the years 1997 and 2002. The dependent variable is a dummy equal to one if the individual supports an active role of the state. The right-hand side variables of interest are the *East* dummy and the interaction terms between *East* and the years 2002 and 2017.

**Table 1:** Baseline regressions.

	Dependent variable: responsibility for financial security…				
	…when	…when	…of the	…when	…when requiring
	unemployed	sick	family	old	care
East	0.438^***^	0.465^***^	0.420^***^	0.436^***^	0.377^***^
	(0.030)	(0.028)	(0.028)	(0.028)	(0.028)
Year 2002	0.043^**^	0.161^***^	−0.004	−0.023	0.113^***^
	(0.020)	(0.020)	(0.021)	(0.020)	(0.020)
East*2002	−0.137^***^	−0.170^***^	−0.097^***^	−0.157^***^	−0.161^***^
	(0.035)	(0.033)	(0.033)	(0.032)	(0.033)
Year 2017	0.202^***^	0.275^***^	0.130^***^	0.153^***^	0.267^***^
	(0.024)	(0.024)	(0.024)	(0.024)	(0.023)
East*2017	−0.333^***^	−0.207^***^	−0.119^***^	−0.182^***^	−0.162^***^
	(0.040)	(0.038)	(0.038)	(0.038)	(0.038)
Age	−0.020^*^	0.023^**^	−0.009	0.009	0.009
	(0.011)	(0.011)	(0.011)	(0.011)	(0.011)
Age squared (*10^3^)	0.495^**^	−0.373^*^	0.216	−0.149	−0.214
	(0.214)	(0.207)	(0.211)	(0.207)	(0.205)
Age cubed (*10^5^)	−0.337^***^	0.190	−0.158	0.078	0.120
	(0.129)	(0.125)	(0.128)	(0.125)	(0.124)
College	−0.193^***^	−0.297^***^	−0.221^***^	−0.316^***^	−0.126^**^
	(0.064)	(0.059)	(0.059)	(0.059)	(0.058)
Vocational training	−0.157^**^	−0.221^***^	−0.249^***^	−0.226^***^	−0.105^*^
	(0.061)	(0.056)	(0.057)	(0.057)	(0.056)
Secondary schooling	−0.160^**^	−0.147^**^	−0.151^**^	−0.132^**^	−0.051
	(0.063)	(0.058)	(0.059)	(0.058)	(0.058)
Intermediate schooling	−0.181^***^	−0.191^***^	−0.268^***^	−0.178^***^	−0.118^*^
	(0.069)	(0.065)	(0.066)	(0.065)	(0.064)
Male	−0.039^**^	−0.046^***^	−0.015	0.002	0.027^*^
	(0.015)	(0.015)	(0.015)	(0.015)	(0.015)
Number of children	0.046^***^	0.038^***^	0.072^***^	0.029^***^	0.017^*^
	(0.011)	(0.010)	(0.010)	(0.010)	(0.010)
Number of adults	0.023^***^	0.028^***^	0.021^***^	0.020^***^	0.009^**^
	(0.004)	(0.004)	(0.004)	(0.004)	(0.004)
Married	0.026	0.050^**^	0.025	0.043^*^	0.045^*^
	(0.026)	(0.025)	(0.025)	(0.025)	(0.025)
Divorced	0.007	−0.032	0.023	−0.000	0.003
	(0.035)	(0.034)	(0.034)	(0.034)	(0.033)
Married but separated	−0.022	−0.010	0.001	0.020	0.050
	(0.053)	(0.051)	(0.052)	(0.052)	(0.051)
Widowed	0.011	0.013	0.010	0.019	0.033
	(0.039)	(0.038)	(0.038)	(0.037)	(0.037)
Log HH income	−0.149^***^	−0.209^***^	−0.169^***^	−0.239^***^	−0.168^***^
	(0.017)	(0.016)	(0.016)	(0.016)	(0.016)
Civil servant	−0.189^***^	−0.222^***^	0.049	−0.116^***^	−0.168^***^
	(0.040)	(0.041)	(0.041)	(0.041)	(0.039)
Self-employed	−0.324^***^	−0.328^***^	−0.266^***^	−0.394^***^	−0.241^***^
	(0.035)	(0.036)	(0.037)	(0.036)	(0.035)
White-collar worker	−0.046^*^	−0.048^**^	−0.025	−0.101^***^	−0.076^***^
	(0.023)	(0.022)	(0.023)	(0.022)	(0.022)
Unemployed	0.122^***^	0.002	0.117^***^	0.000	−0.015
	(0.039)	(0.036)	(0.036)	(0.036)	(0.036)
Retired	−0.125^***^	−0.113^***^	0.005	−0.069^*^	−0.031
	(0.038)	(0.036)	(0.037)	(0.036)	(0.036)
Maternity	0.002	−0.072	0.016	−0.150^**^	−0.069
	(0.061)	(0.058)	(0.058)	(0.058)	(0.058)
Nonworking	−0.038	−0.035	0.060**	−0.030	−0.019
	(0.030)	(0.029)	(0.029)	(0.029)	(0.029)
Training	−0.093	−0.066	−0.203^***^	−0.071	−0.065
	(0.059)	(0.057)	(0.058)	(0.057)	(0.056)
Other nonworking	−0.014	−0.075^**^	−0.003	−0.050	−0.095^**^
	(0.040)	(0.038)	(0.038)	(0.038)	(0.038)
Observations	36,564	36,599	36,532	36,636	36,637
Individuals	24,819	24,840	24,801	24,847	24,848
Log likelihood	−22,180	−24,421	−23,638	−24,330	−24,961

Probit regressions. The dependent variable is an indicator variable that takes the value one if the household responds *only the state* or *mostly the state* to the question of who should be responsible for the financial security of different groups. Omitted categories are fewer than nine years of schooling, female, single, blue-collar worker, and employed. ^*^ *p* < 0.10, ^**^ *p* < 0.05, ^***^ *p* < 0.01. Robust standard errors clustered on individual level in parentheses.

As Table 1 shows, individuals who lived in East Germany before reunification have consistently and significantly stronger pro-state preferences than individuals from West Germany in 1997, as expressed by the positive and significant coefficients on the *East* dummy. Having lived under different systems for up to 45 years shaped the preferences of East and West Germans. Moreover, the preferences of East and West Germans converge significantly between 1997 and 2002 – the coefficients of the interaction terms East*2002 are all significantly negative. This is in line with a common effect on preferences from living under the same system since 1990. The results so far are also quantitatively very similar to the results in Alesina and Fuchs-Schündeln (2007). Extending the time frame to include the 2017 survey has thus no effect on the results from the previous years.

What is interesting, though, is how preferences evolved between 2002 and 2017. First focusing on West Germans, there was already some movement toward stronger state preferences between 1997 and 2002 – the *Year 2002* dummy is positive and significant in 3 out of 5 cases – but this movement became even stronger between 2002 and 2017: the *Year 2017* dummy is positive and significant for all five variables. It is also significantly different from the *Year 2002* dummy (Appendix Table A5). Thus, West German preferences moved more in favor of a strong government in the two decades between 1997 and 2017. Turning to the coefficients on the interaction terms with the East dummy, the preference differences between East and West Germans continued to narrow: the coefficients of East*2017 are not only significantly negative but also larger in absolute magnitude than the East*2002 coefficients. Yet, only in the case of taking care of unemployed individuals is the East*2017 coefficient significantly different from the East*2002 coefficient. Thus, there is significant convergence between 2002 and 2017 only in one of the five preference variables. In the other four cases, the convergence is not only not significant in this period, but according to the point estimates also substantially slower than in the period 1997 to 2002. This is true even for the only variable in which significant convergence still occurs between 2002 and 2017: taking into account that this is a 15-year period, compared to the 5-year period between 1997 and 2002, the speed of convergence slowed down significantly. The coefficients on the control variables are very close to the estimates in Alesina and Fuchs-Schündeln (2007) both in sign and magnitude. Individuals with more education and higher monthly household income, as well as the self-employed and white-collar workers, are consistently less pro-state.4
Appendix Table A6 shows that the main coefficients are quite similar in regressions without any control variables.


The marginal effects corresponding to Table 1 are shown in Appendix Table A7. They allow us to talk about the speed of convergence more concretely. In 1997, individuals from East Germany were between 14.8 and 17.8 percentage points more likely to have pro-government preferences than individuals from West Germany. Compared to 1997, the East-West difference in the probability of being pro-state declined by between 3.6 and 6.5 percentage points in 2002, and by between 4.4 and 11.5 percentage points in 2017. Clearly, the convergence of preferences between East and West Germans is not complete – even 27 years after reunification the preferences regarding the active role of state differ between East and West Germans. If the speed of convergence continues as it did between 2002 and 2017, then preferences for taking care of the unemployed should converge by 2025. However, for the other three variables which still show convergence between 2002 and 2017, final convergence of preferences would take significantly longer. In the case of taking care of the sick, for instance, it will take a further 98 years after 2017 for preferences to converge; both because the difference is still quite large in 2017 (East Germans are 9.8 percentage points more likely to answer that the state should take care of sick individuals), and because convergence is slow (the East-West difference declined by only 1.5 percentage points over the 15 year period 2002 to 2017). For the other two variables, the much lower speed of convergence between 2002 and 2017 implies that full convergence of preferences will take between 144 (taking care of old individuals) and 208 years (taking care of individuals requiring care). Thus, not only is the convergence between 2002 and 2017 not statistically significant, neither is it economically significant.

Individual preferences for a strong government could be affected not only by the individual economic situation, but also by general economic circumstances, either because individuals are altruistic and care for people around them, or because they could potentially be affected by these general economic circumstances themselves in the future. Thus, we include as an additional control variable the state-level unemployment rate in the survey year.5The state-level unemployment rate is obtained from the Federal Employment Agency. As Table 2 shows, indeed individuals who reside in states with higher unemployment rates tend to exhibit stronger preferences for an active role of the state: the coefficients on the state-level unemployment rate are positive and statistically significant across all five dependent variables. The inclusion of this control variable decreases the magnitude of the *East* coefficients, which suggests that part of the *East* effect can be explained by higher unemployment rates in the Eastern states. Nevertheless, the *East* dummy is still a significant predictor of preferences. The convergence results are also quantitatively affected by the inclusion of this control variable: the inclusion of the state-level unemployment rate leads to stronger convergence between 1997 and 2002, and weaker convergence or even divergence between 2002 and 2017.

**Table 2:** Regressions controlling for state-level unemployment rate.

	Dependent variable: responsibility for financial security…				
	…when	…when	…of the	…when	…when requiring
	unemployed	sick	family	old	care
East	0.292^***^	0.359^***^	0.311^***^	0.330^***^	0.258^***^
	(0.039)	(0.036)	(0.036)	(0.036)	(0.036)
Year 2002	0.079^***^	0.187^***^	0.023	0.003	0.142^***^
	(0.021)	(0.021)	(0.021)	(0.021)	(0.021)
East*2002	−0.161^***^	−0.187^***^	−0.115^***^	−0.175^***^	−0.181^***^
	(0.036)	(0.033)	(0.033)	(0.033)	(0.033)
Year 2017	0.291^***^	0.339^***^	0.195^***^	0.217^***^	0.339^***^
	(0.028)	(0.027)	(0.028)	(0.027)	(0.027)
East*2017	−0.217^***^	−0.122^***^	−0.032	−0.098^**^	−0.067
	(0.045)	(0.042)	(0.042)	(0.042)	(0.042)
Regional unemployment rate	0.019^***^	0.014^***^	0.014^***^	0.014^***^	0.016^***^
	(0.003)	(0.003)	(0.003)	(0.003)	(0.003)
All control variables	Yes	Yes	Yes	Yes	Yes
Observations	36,563	36,598	36,531	36,635	36,636
Individuals	24,818	24,839	24,800	24,846	24,847
Log likelihood	−22,160	−24,409	−23,625	−24,318	−24,946

Probit regressions. The dependent variable is an indicator variable that takes the value one if the household responds *only the state* or *mostly the state* to the question of who should be responsible for the financial security of different groups. Included as controls are cubic function in age, number of children and number of adults in household, logarithm of household income, and dummies for education, sex, marital status, employment status, and occupation. ^*^ *p* < 0.10, ^**^ *p* < 0.05, ^***^ *p* < 0.01. Robust standard errors clustered on individual level in parentheses.

In Table 3, we split the Eastern sample into East German states by current residence, as in Table 8 of Alesina and Fuchs-Schündeln (2007). We drop all individuals who are originally from the East and now reside in a West state and vice versa. The table shows that the patterns we found before are quite homogeneous and are thus observed across individual states as well. First, in 1997 individuals from all Eastern states are significantly more pro-state than their West German counterparts. Secondly, all the significant coefficients of the interaction terms measuring convergence are negative (except for one coefficient for Brandenburg in 2002 and one for Mecklenburg-Vorpommern in 2017). For 2002, 15 of the 25 convergence coefficients are negative and significant, and the same is true for 2017. Convergence is stronger in Sachsen-Anhalt, Thüringen, and Sachsen than in the other three states. Generally, this analysis confirms that the baseline results are not driven by a single state.

**Table 3:** Splitting East into states, unbalanced sample.

	Dependent variable: responsibility for financial security…				
	…when	…when	…of the	…when	…when requiring
	unemployed	sick	family	old	care
Mecklenburg-Vorpommern	0.300^***^	0.337^***^	0.229^***^	0.305^***^	0.401^***^
	(0.076)	(0.070)	(0.070)	(0.070)	(0.070)
Brandenburg	0.407^***^	0.380^***^	0.416^***^	0.462^***^	0.404^***^
	(0.063)	(0.058)	(0.057)	(0.058)	(0.058)
Sachsen-Anhalt	0.502^***^	0.585^***^	0.464^***^	0.581^***^	0.462^***^
	(0.062)	(0.055)	(0.055)	(0.055)	(0.055)
Thüringen	0.611^***^	0.451^***^	0.441^***^	0.444^***^	0.372^***^
	(0.064)	(0.054)	(0.054)	(0.055)	(0.055)
Sachsen	0.478^***^	0.536^***^	0.477^***^	0.456^***^	0.376^***^
	(0.049)	(0.044)	(0.044)	(0.044)	(0.044)
Year 2002	0.046^**^	0.154^***^	0.000	−0.022	0.115^***^
	(0.020)	(0.020)	(0.021)	(0.020)	(0.020)
Mecklenburg-Vorpommern*2002	0.003	0.008	−0.002	−0.068	−0.172^*^
	(0.100)	(0.092)	(0.089)	(0.088)	(0.090)
Brandenburg*2002	0.145^*^	0.093	0.078	−0.011	−0.064
	(0.080)	(0.070)	(0.068)	(0.071)	(0.071)
Sachsen-Anhalt*2002	−0.156^**^	−0.276^***^	−0.146^**^	−0.319^***^	−0.289^***^
	(0.074)	(0.065)	(0.066)	(0.065)	(0.066)
Thüringen*2002	−0.544^***^	−0.181^***^	−0.217^***^	−0.141^**^	−0.063
	(0.073)	(0.066)	(0.065)	(0.066)	(0.064)
Sachsen*2002	−0.136^**^	−0.225^***^	−0.155^***^	−0.200^***^	−0.162^***^
	(0.058)	(0.053)	(0.052)	(0.052)	(0.053)
Year 2017	0.198^***^	0.259^***^	0.127^***^	0.156^***^	0.269^***^
	(0.024)	(0.024)	(0.024)	(0.024)	(0.024)
Mecklenburg-Vorpommern*2017	−0.192^*^	−0.084	0.183^*^	−0.090	−0.073
	(0.109)	(0.103)	(0.101)	(0.101)	(0.105)
Brandenburg*2017	−0.104	−0.013	0.028	−0.121	−0.069
	(0.090)	(0.080)	(0.081)	(0.082)	(0.082)
Sachsen-Anhalt*2017	−0.326^***^	−0.283^***^	−0.200^**^	−0.223^***^	−0.130
	(0.091)	(0.080)	(0.079)	(0.079)	(0.081)
Thüringen*2017	−0.829^***^	−0.367^***^	−0.369^***^	−0.478^***^	−0.346^***^
	(0.084)	(0.078)	(0.078)	(0.079)	(0.079)
Sachsen*2017	−0.298^***^	−0.165^***^	−0.113^*^	−0.109^*^	−0.161^***^
	(0.069)	(0.063)	(0.062)	(0.062)	(0.062)
All control variables	Yes	Yes	Yes	Yes	Yes
Observations	33,845	33,887	33,825	33,916	33,915
Individuals	23,048	23,070	23,036	23,076	23,077
Log likelihood	−20,545	−22,547	−21,777	−22,452	−23,057

Probit regressions. The dependent variable is an indicator variable that takes the value one if the household responds *only the state* or *mostly the state* to the question of who should be responsible for the financial security of different groups. Included as controls are cubic function in age, number of children and number of adults in household, logarithm of household income, and dummies for education, sex, marital status, employment status, and occupation. ^*^ *p* < 0.10, ^**^ *p* < 0.05, ^***^ *p* < 0.01. Robust standard errors clustered on individual level in parentheses.

### 3.1 Heterogeneity by Cohort

Table 4 replicates the results presented in Table 3 of Alesina and Fuchs-Schündeln (2007), in which we allow for separate cohort patterns in East and West.6
Appendix Table A10 replicates Table 2 of Alesina and Fuchs-Schündeln (2007), which allows instead for separate age patterns in East and West. As in Alesina and Fuchs-Schündeln (2007), we find that older cohorts from the East are significantly more pro-state compared to younger cohorts, while the opposite is true for West Germans. Thus, the East-West differences in preferences are increasing in the time the individuals lived under separate regimes. This is convincing evidence of the effect of the regime on preferences.

**Table 4:** Regressions with cohorts interacted with East.

	Dependent variable: responsibility for financial security…				
	…when	…when	…of the	…when	…when requiring
	unemployed	sick	family	old	care
East	0.295^***^	0.284^***^	0.231^***^	0.187^***^	0.214^***^
	(0.053)	(0.049)	(0.050)	(0.050)	(0.050)
Year 2002	0.036^*^	0.133^***^	−0.035	−0.062^***^	0.086^***^
	(0.021)	(0.021)	(0.022)	(0.021)	(0.021)
East*2002	−0.133^***^	−0.159^***^	−0.086^***^	−0.146^***^	−0.152^***^
	(0.036)	(0.033)	(0.033)	(0.033)	(0.033)
Year 2017	0.183^***^	0.184^***^	0.022	0.027	0.170^***^
	(0.035)	(0.034)	(0.035)	(0.034)	(0.033)
East*2017	−0.304^***^	−0.169^***^	−0.076^**^	−0.130^***^	−0.125^***^
	(0.041)	(0.038)	(0.038)	(0.038)	(0.038)
Born 1961–1975	−0.042	−0.185^***^	−0.215^***^	−0.274^***^	−0.155^***^
	(0.040)	(0.038)	(0.039)	(0.039)	(0.038)
Born 1946–1960	−0.123^**^	−0.336^***^	−0.336^***^	−0.435^***^	−0.285^***^
	(0.055)	(0.053)	(0.054)	(0.053)	(0.052)
Born before 1946	−0.137^*^	−0.358^***^	−0.420^***^	−0.513^***^	−0.367^***^
	(0.074)	(0.072)	(0.073)	(0.072)	(0.071)
Born 1961–1975*East	−0.047	0.050	0.047	0.053	0.030
	(0.054)	(0.050)	(0.051)	(0.051)	(0.050)
Born 1946–1960*East	0.164^***^	0.160^***^	0.148^***^	0.206^***^	0.128^**^
	(0.054)	(0.050)	(0.051)	(0.051)	(0.050)
Born before 1946*East	0.339^***^	0.347^***^	0.377^***^	0.505^***^	0.341^***^
	(0.054)	(0.050)	(0.051)	(0.051)	(0.050)
All control variables	Yes	Yes	Yes	Yes	Yes
Observations	36,564	36,599	36,532	36,636	36,637
Individuals	24,819	24,840	24,801	24,847	24,848
Log likelihood	−22,126	−24,353	−23,565	−24,196	−24,902

Probit regressions. The dependent variable is an indicator variable that takes the value one if the household responds *only the state* or *mostly the state* to the question of who should be responsible for the financial security of different groups. Included as controls are cubic function in age, number of children and number of adults in household, logarithm of household income, and dummies for education, sex, marital status, employment status, and occupation. The omitted category is *Born after 1989*. ^*^ *p* < 0.10, ^**^ *p* < 0.05, ^***^ *p* < 0.01. Robust standard errors clustered on individual level in parentheses.

### 3.2 Results for Balanced Sample

Finally, Table 5 again builds on an exercise by Alesina and Fuchs-Schündeln (2007). Here, we restrict the sample to a balanced sample. This allows us to understand whether the shift in state-related preferences is driven by the change in the cohort composition of the sample, or whether it occurs on the individual level. Given that younger cohorts have more similar preferences than older cohorts, some convergence will naturally occur over time as the relative role of younger cohorts increases. This effect is controlled for if we only include individuals whom we observe in all sample years. Since here we include only individuals who are observed over two decades, the sample size drops from around 36,500 observations to only roughly 7000 observations.

**Table 5:** Balanced sample: regressions with individuals who answer in 1997, 2002 and 2017.

	Dependent variable: responsibility for financial security…				
	…when	…when	…of the	…when	…when requiring
	unemployed	sick	family	old	care
East	0.412^***^	0.401^***^	0.402^***^	0.280^***^	0.300^***^
	(0.060)	(0.057)	(0.057)	(0.057)	(0.057)
Year 2002	0.070	0.194^***^	0.005	−0.010	0.192^***^
	(0.046)	(0.045)	(0.047)	(0.046)	(0.045)
East*2002	−0.143^**^	−0.139^**^	−0.024	−0.027	−0.157^**^
	(0.073)	(0.069)	(0.070)	(0.067)	(0.068)
Year 2017	0.144^**^	0.319^***^	0.096^*^	0.159^***^	0.340^***^
	(0.057)	(0.054)	(0.056)	(0.056)	(0.055)
East*2017	−0.157^**^	−0.165^**^	−0.031	0.037	−0.162^**^
	(0.079)	(0.073)	(0.071)	(0.072)	(0.074)
All control variables	Yes	Yes	Yes	Yes	Yes
Observations	6981	6981	6981	6981	6981
Individuals	2327	2327	2327	2327	2327
Log likelihood	−4122	−4651	−4534	−4627	−4744

Probit regressions. The dependent variable is an indicator variable that takes the value one if the household responds *only the state* or *mostly the state* to the question of who should be responsible for the financial security of different groups. Included as controls are cubic function in age, number of children and number of adults in household, logarithm of household income, and dummies for education, sex, marital status, employment status, and occupation. ^*^ *p* < 0.10, ^**^ *p* < 0.05, ^***^ *p* < 0.01. Robust standard errors clustered on individual level in parentheses.

The results based on the balanced sample remain qualitatively unchanged: Individuals from East Germany have significantly stronger preferences for an active role of the state in providing financial security, and the East-West difference in preferences becomes smaller over time. Thus, the convergence of preferences between East and West is not explained by a shift in the cohort composition alone. Similar to the unbalanced sample, the pace of convergence is faster between 1997 and 2002 and is heterogeneous across the dimensions of preferences. In the balanced sample, we still observe convergence of preferences between 2002 and 2017 in all dimensions but taking care of the old, but this convergence is never significant: none of the coefficients on the interactions of East*2002 and East*2017 are significantly different.

Looking at the marginal effects across the two samples (Appendix Tables A7 and A8), we notice that the estimated convergence between 2002 and 2017 is in fact quite a bit faster in the unbalanced sample. For instance, the probability of being pro-state when it comes to the role of the state in case of unemployment decreased by 6.8 percentage points in 2017 compared to 2002 in the unbalanced sample, but only by 0.5 percentage points in the balanced sample. This means that the overall convergence between 2002 and 2017 is driven mainly by the change in the composition of the sample. The coefficient of *Year 2017* is significant and positive in the balanced sample as well – West Germans became more pro-state compared to both 1997 and 2002. Relative to the West Germans, there is no significant development of East German preferences away from the state.

One might be concerned that selective attrition drives the differences in speed of convergence between the balanced and unbalanced samples. In Appendix Table A9, we test whether the probability of staying in the sample between two consecutive sample periods (1997 and 2002 or 2002 and 2017, respectively) is correlated with the preferences in the first of the two respective sample periods. We find that there is no systematic pattern, with five coefficients being positive and five negative, and only three of the ten coefficients – one positive and two negative – being statistically significant. Thus, the differences in convergence results between the two samples do not stem from selective attrition.

### 3.3 Heterogenity by Former Support of Regime

In 2018, SOEP added a special module on “Life in the former GDR”. This module, administered only to respondents who grew up in the East, asks several questions related to their life in the former GDR. While there might be a recollection bias in answering these questions 30 years later, we still use this information to give some suggestive evidence on whether former closeness to the regime is related to preferences and their convergence. To do this, we follow Deter and Lange (2022) and split the Eastern sample into three groups based on the level of conformism to the GDR political regime. Specifically, we define respondents as *supporters* of the regime if they were members of the SED party or worked in the sensitive public sector. *Opponents* of the regime are those who participated in the demonstrations and opposition movements in 1989 and 1990 (and were in addition not previously assigned to the supporter group). The rest of the East sample are the *silent majority*. Similarly to Deter and Lange (2022), supporters and opponents constitute 20% and 22% of the Eastern sample, respectively. Note that, rather than roughly 12,500 East German observations in our baseline sample, we have only 4220 observations of East Germans here, since many respondents did not answer this module (as in the balanced sample, we lose a lot of East observations due to attrition).

Table 6 shows the results if we split the East German sample into the three groups of supporters, opponents, and silent majority. In 1997, all three Eastern groups were significantly more pro-government than their Western peers: all the coefficients are positive, and except for one also statistically significant. The supporters of the Communist regime tend to exhibit the strongest pro-state preferences in 1997, and in two cases – financial security of families and of those requiring care – their coefficients are significantly larger than the ones of the opponents. Convergence of preferences happened for all three groups, but tended to happen later for the supporters and the silent majority than for the opponents. Thus, it seems that initial preference differences were larger between West Germans and those East Germans who supported the regime, and convergence also took more time between these two groups than between West Germans and the rest of the East Germans.

**Table 6:** Splitting the East sample by support of the regime.

	Dependent variable: responsibility for financial security…				
	…when	…when	…of the	…when	…when requiring
	unemployed	sick	family	old	care
Supporter	0.569^***^	0.551^***^	0.644^***^	0.309^***^	0.406^***^
	(0.120)	(0.104)	(0.103)	(0.104)	(0.104)
Silent majority	0.487^***^	0.456^***^	0.357^***^	0.384^***^	0.347^***^
	(0.072)	(0.063)	(0.063)	(0.063)	(0.063)
Opponent	0.394^***^	0.379^***^	0.404^***^	0.293^***^	0.125
	(0.102)	(0.093)	(0.094)	(0.093)	(0.093)
Supporter*2002	−0.195	−0.194	−0.257^**^	0.021	−0.102
	(0.140)	(0.124)	(0.113)	(0.113)	(0.116)
Supporter*2017	−0.420^***^	−0.370^***^	−0.211^*^	−0.005	−0.177
	(0.138)	(0.114)	(0.114)	(0.119)	(0.120)
Opponent*2002	−0.229^*^	−0.223^**^	−0.127	−0.048	0.020
	(0.122)	(0.111)	(0.115)	(0.110)	(0.110)
Opponent*2017	−0.184	0.002	−0.147	−0.095	0.125
	(0.117)	(0.107)	(0.106)	(0.108)	(0.109)
Silent majority*2002	−0.141^*^	−0.131^*^	−0.035	−0.086	−0.116
	(0.084)	(0.074)	(0.074)	(0.072)	(0.072)
Silent majority*2017	−0.369^***^	−0.194^***^	−0.042	−0.153^**^	−0.138^*^
	(0.082)	(0.072)	(0.072)	(0.071)	(0.073)
Year 2002	0.051^**^	0.167^***^	0.004	−0.014	0.119^***^
	(0.020)	(0.020)	(0.021)	(0.020)	(0.020)
Year 2017	0.228^***^	0.308^***^	0.157^***^	0.194^***^	0.295^***^
	(0.024)	(0.024)	(0.024)	(0.024)	(0.024)
All control variables	Yes	Yes	Yes	Yes	Yes
Observations	28,727	28,758	28,693	28,782	28,780
Individuals	19,350	19,372	19,339	19,373	19,373
Log likelihood	−17,935	−19,025	−18,239	−18,962	−19,574

Probit regressions. The dependent variable is an indicator variable that takes the value one if the household responds *only the state* or *mostly the state* to the question of who should be responsible for the financial security of different groups. Included as controls are cubic function in age, number of children and number of adults in household, logarithm of household income, and dummies for education, sex, marital status, employment status, and occupation. ^*^ *p* < 0.10, ^**^ *p* < 0.05, ^***^ *p* < 0.01. Robust standard errors clustered on individual level in parentheses.

Generally, our analyses confirm the results presented in Alesina and Fuchs-Schündeln (2007). As the German reunification experiment shows, political systems shape individual preferences toward the role of government. People subjected to up to 45 years of Communism in East Germany exhibit views in favor of an active role of the state in providing financial security for vulnerable groups. A caveat of the analysis is that we can observe preferences for the first time in 1997, i.e. 7 years after reunification. The experience in the transition period, e.g. the rapid privatization (see Mergele et al. 2020), the increase in unemployment, and the increase in inequality (see Fuchs-Schündeln et al. 2010), could have affected preferences as well. We control for this to the extent possible by controlling for individual economic circumstances, as well as state-level unemployment rates in Table 2. A novel result compared to Alesina and Fuchs-Schündeln (2007) is that the speed of convergence of East-West preferences decreased substantially over time. While in the initial period after reunification preferences converged rapidly, the remaining convergence in the last 15 years is almost exclusively driven by changes in the cohort composition. At the same time, the preferences of West Germans shifted towards state-provided social security. Given the uneven pace of convergence, it seems unlikely that the difference in preferences will disappear anytime soon.

## 4 Preferences of Post-Reunification Cohorts

So far, we find that convergence of preferences among individuals who were born before reunification significantly slowed down between 2002 and 2017. However, should we expect faster convergence once younger individuals born after reunification and thus never having accumulated different experiences enter the sample?

We analyze this question explicitly by focusing on the cohorts born between 1990 and 1999. These cohorts were born after the Fall of the Berlin Wall and never experienced German separation. Table 7 presents the results of the regression analysis based on the sample of these young individuals in 2017. There are two regressors of interest: *Born in East States* is a dummy variable equal to one if the respondent was born in one of the states that formerly belonged to East Germany; *Born to Eastern parents* is a dummy variable equal to one if at least one of the parents of the respondent lived in the GDR prior to 1989. Given the small number of observations and the young age of this sample, the control variables only include age, gender, and labor market variables (with *Employed* as the omitted category).

**Table 7:** Regressions with those who were born in 1990–1999.

	Dependent variable: responsibility for financial security…				
	…when	…when	…of the	…when	…when requiring
	unemployed	sick	family	old	care
Born in East states	−0.118	0.075	−0.118	0.043	0.027
	(0.198)	(0.187)	(0.183)	(0.186)	(0.184)
Born to Eastern parents	0.145	0.062	0.388^**^	0.357^*^	0.121
	(0.195)	(0.184)	(0.181)	(0.183)	(0.181)
Age	0.009	0.003	0.013	0.028^*^	0.045^***^
	(0.016)	(0.015)	(0.016)	(0.016)	(0.016)
Male	−0.066	−0.008	0.084	−0.074	−0.035
	(0.090)	(0.087)	(0.087)	(0.087)	(0.087)
Unemployed	0.511^*^	0.041	−0.098	−0.030	−0.057
	(0.289)	(0.249)	(0.242)	(0.251)	(0.248)
Maternity	0.685	0.598	−0.573	−0.223	0.423
	(0.573)	(0.487)	(0.500)	(0.450)	(0.494)
Training	0.198^*^	0.114	−0.018	0.051	0.134
	(0.111)	(0.106)	(0.105)	(0.106)	(0.106)
Other nonworking	0.428^***^	0.283^*^	0.129	0.190	0.031
	(0.164)	(0.152)	(0.152)	(0.151)	(0.150)
Observations	867	864	868	866	866
Log likelihood	−530	−591	−589	−588	−587

Probit regressions. The dependent variable is an indicator variable that takes the value one if the household responds *only the state* or *mostly the state* to the question of who should be responsible for the financial security of different groups. ^*^ *p* < 0.10, ^**^ *p* < 0.05, ^***^ *p* < 0.01. Robust standard errors in parentheses.

The results presented in Table 7 refute the ex-ante assumption that East and West Germans born after reunification do not exhibit any difference in preferences. Individuals born to “East” parents in the period 1990 to 1999 exhibit stronger pro-state preferences compared to their Western counterparts, even though they have never lived under Communism themselves; two of the relevant coefficients are also statistically significant. By contrast, the coefficients on the dummy variable *Born in East States* are small, never significant, and sometimes even negative. This indicates that parents might be more important than peers or the general environment in shaping preferences. Although never directly affected by Communism themselves, respondents are more likely to support the responsibility of the state if at least one of their parents lived under Communism.

Table 8 sheds some more light on this result. In this estimation, we include the parental preferences directly as a control variable. Specifically, we create a dummy variable *Parent pro-state in 2017* equal to one if at least one of the individual’s parents – mother or father – answered *only the state* or *mostly the state* to the question about the state’s responsibilities in providing financial security in 2017. The coefficient of this dummy variable is positive and statistically significant in all regressions: parents’ preferences are strongly correlated with a young person’s attitudes. This is true for both East and West Germans. At the same time, the coefficient of *Born to Eastern parents* is now somewhat smaller in magnitude in four of the five regressions than in Appendix Table A11, which shows the results of a regression without parental preferences on exactly the same sample (which is somewhat smaller than the sample in Table 7). This suggests that the variable *Born to Eastern parents* acts to a certain extent as a proxy for the previously unobserved parental preferences. Respondents with “East” parents are more likely to have parents with strong pro-state preferences, and the intergenerational transmission of preferences leads to differences in preferences also among the cohorts born after reunification. Appendix Tables A12 and A13 confirm that preferences of both parents matter. Fathers’ attitudes are equally important as mothers’ attitudes – all the coefficients of *Mother pro-state in 2017* and *Father pro-state in 2017* are large and statistically significant.

**Table 8:** Regressions with those who were born in 1990–1999, including parents’ preferences.

	Dependent variable: responsibility for financial security…				
	…when	…when	…of the	…when	…when requiring
	unemployed	sick	family	old	care
Born in East states	−0.199	0.011	−0.190	−0.021	−0.059
	(0.204)	(0.195)	(0.189)	(0.193)	(0.192)
Born to Eastern parents	0.228	0.094	0.418^**^	0.383^**^	0.161
	(0.202)	(0.193)	(0.187)	(0.190)	(0.189)
Parent pro-state in 2017	0.470^***^	0.195^**^	0.410^***^	0.288^***^	0.230^**^
	(0.114)	(0.090)	(0.088)	(0.088)	(0.093)
Age	0.005	0.003	0.014	0.023	0.042^***^
	(0.017)	(0.016)	(0.016)	(0.016)	(0.016)
Male	−0.050	−0.005	0.070	−0.098	−0.052
	(0.092)	(0.088)	(0.089)	(0.088)	(0.088)
Unemployed	0.404	0.120	−0.050	−0.068	−0.098
	(0.306)	(0.264)	(0.257)	(0.266)	(0.264)
Maternity	0.577	0.967^*^	−0.603	−0.025	0.787
	(0.610)	(0.575)	(0.517)	(0.496)	(0.624)
Training	0.203^*^	0.088	−0.049	0.049	0.116
	(0.112)	(0.106)	(0.107)	(0.106)	(0.107)
Other nonworking	0.453^***^	0.289^*^	0.113	0.168	0.006
	(0.166)	(0.154)	(0.157)	(0.153)	(0.152)
Observations	846	843	844	845	846
Log likelihood	−510	−572	−562	−569	−569

Probit regressions. The dependent variable is an indicator variable that takes the value one if the household responds *only the state* or *mostly the state* to the question of who should be responsible for the financial security of different groups. ^*^ *p* < 0.10, ^**^ *p* < 0.05, ^***^ *p* < 0.01. Robust standard errors in parentheses.

Summarizing, young cohorts born between 1990 and 1999 exhibit stronger pro-state preferences if at least one of their parents lived in the GDR. They are significantly more likely to hold the view that it is the state’s responsibility to provide financial security for families and older individuals. However, the pro-state attitudes of these cohorts are no longer driven by the Communist context during their upbringing, as they were born in a united Germany, but by the pro-state views of their parents, who were subject to this context in the past. The experience of the parents matters more than the experience of the peers, since growing up in an Eastern or Western state after reunification plays no significant role for preferences. A similar transmission of preferences from parents to children has been shown in other contexts, for example when it comes to the support of right-wing extremism (see Avdeenko and Siedler 2017).

## 5 Conclusion

In this paper, we investigate the influence of the political system on preferences about the role of the government. The German reunification experiment provides an ideal setting for such an investigation, as it constitutes an exogenous shock to the political system. East Germans spent several decades under the Communist regime, being exposed to its doctrine and values. After reunification in 1990, Communism gave way to a market-based economy. Under the assumption that there were no meaningful differences between the residents of East and West Germany before the separation, the differences in preferences that we observe after reunification can be attributed to living under the Communist regime. Even if there were differences between East and West before the separation, convergence in preferences on the individual level after reunification, as well as stronger differences in preferences for cohorts that lived under different systems for longer, point toward the effect of Communism.

Alesina and Fuchs-Schündeln (2007) quantify the East-West differences in preferences after reunification, using data from the German SOEP until 2002. Here, we replicate and extend their findings, adding the 2017 data wave. We find that even 27 years after reunification, East Germans remain more pro-state than West Germans. Our analysis suggests that in 1997 they are more likely to state it is the government’s responsibility to provide financial security for individuals who are sick, unemployed, old, or in need of care, as well as for families, compared to their West German peers. We also observe that preferences converge and adjust over time, with East Germans becoming relatively less pro-state, and West Germans appreciating the welfare state more than before. However, the pace of convergence is not uniform. The convergence of preferences was rapid in the first years after reunification, but slowed down significantly after 2002. Most of the remaining convergence comes from shifts in the cohort composition, not from convergence of preferences on the individual level.

How do preferences of individuals born *after* reunification compare between East and West? We find that young individuals born after reunification exhibit stronger preferences for a strong government if at least one of their parents lived in the GDR. The former place of living of the parents matters more than the current place of living of the young individual for his or her preferences. It seems that through intergenerational transmission of preferences the effects of Communism are thus truly long-lasting.

## Supplementary Material

Supplementary Material Details
