# Supplementary material for: Good Bye Lenin Revisited: East-West Preferences Three Decades after German Reunification
Source: Ger Econ Rev. 2023 Feb 24;24(1):97–119. doi: 10.1515/ger-2022-0042 (PMC10005892; doi:10.1515/ger-2022-0042)
Supplement: Supplementary file 1 — Supplementary Material Details [file j_ger-2022-0042_suppl.pdf]

Online Appendix

Good Bye Lenin Revisited: East-West Preferences Three Decades After German Reunification

Mariia Bondar, Charles River Associates

Nicola Fuchs-Schündeln, Goethe University Frankfurt and CEPR

January 29, 2023

Summary statistics

Table A1: Mean of the dependent variables, by East/West and Year, unbalanced sample

	West		East	
	Mean	N	Mean	N
1997				
State responsibility when unemployed	0.63	6094	0.79	3680
...when sick	0.35	6097	0.53	3674
...of the family	0.33	6087	0.49	3679
...when old	0.39	6100	0.56	3685
...when requiring care	0.41	6099	0.57	3685
2002				
State responsibility when unemployed	0.64	12474	0.76	5304
...when sick	0.39	12488	0.52	5308
...of the family	0.32	12473	0.45	5300
...when old	0.36	12494	0.49	5313
...when requiring care	0.44	12486	0.54	5318
2017				
State responsibility when unemployed	0.69	7872	0.74	3355
...when sick	0.42	7890	0.54	3363
...of the family	0.35	7860	0.48	3344
...when old	0.41	7898	0.53	3368
...when requiring care	0.48	7899	0.59	3369

Table A2: Mean of the dependent variables, by East/West and Year, balanced sample

	West		East	
	Mean	N	Mean	N
1997				
...when unemployed	0.63	1390	0.78	937
...when sick	0.33	1390	0.49	937
...of the family	0.31	1390	0.47	937
...when old	0.36	1390	0.49	937
...when requiring care	0.39	1390	0.53	937
2002				
...when unemployed	0.66	1390	0.76	937
...when sick	0.40	1390	0.51	937
...of the family	0.32	1390	0.46	937
...when old	0.35	1390	0.47	937
...when requiring care	0.46	1390	0.53	937
2017				
...when unemployed	0.69	1390	0.78	937
...when sick	0.44	1390	0.54	937
...of the family	0.35	1390	0.50	937
...when old	0.41	1390	0.55	937
...when requiring care	0.50	1390	0.57	937

Table A3: Mean of the independent variables, by East/West and Year, unbalanced sample

	West		East	
	Mean	N	Mean	N
1997				
Age	46.36	6164	44.57	3714
College	0.12	6092	0.24	3707
Vocational training	0.63	6111	0.62	3713
Secondary schooling	0.18	6115	0.09	3680
Intermediate schooling	0.04	6164	0.03	3714
Male	0.47	6164	0.48	3714
Number of children	0.53	6164	0.55	3714
Number of adults	3.61	6164	3.05	3714
Married	0.61	6155	0.63	3711
Divorced	0.06	6155	0.06	3711
Married but separated	0.02	6155	0.02	3711
Widowed	0.08	6155	0.06	3711
Log of net HH monthly income, in 2011 Euro	7.90	5891	7.73	3619
Civil servant	0.05	6149	0.02	3710
Self-employed	0.06	6163	0.05	3708
White-collar worker	0.29	6164	0.27	3714
Unemployed	0.04	6164	0.11	3714
Retired	0.16	6164	0.12	3714
Maternity	0.02	6164	0.02	3714
Nonworking	0.13	6164	0.10	3714
Training	0.02	6164	0.02	3714
Other nonworking	0.05	6164	0.05	3714
2002				
Age	48.47	12652	46.78	5380
College	0.15	12579	0.23	5370
Vocational training	0.65	12639	0.64	5379
Secondary schooling	0.13	12580	0.08	5339
Intermediate schooling	0.04	12652	0.03	5380
Male	0.48	12652	0.48	5380
Number of children	0.53	12652	0.39	5380
Number of adults	2.84	12652	2.87	5380
Married	0.63	12641	0.58	5379
Divorced	0.06	12641	0.08	5379
Married but separated	0.02	12641	0.02	5379
Widowed	0.07	12641	0.07	5379
Log of net HH monthly income, in 2011 Euro	7.93	11781	7.75	5200
Civil servant	0.05	12650	0.02	5379
Self-employed	0.06	12652	0.04	5380
White-collar worker	0.29	12642	0.27	5375
Unemployed	0.03	12652	0.09	5380
Retired	0.18	12652	0.16	5380
Maternity	0.02	12652	0.01	5380
Nonworking	0.13	12652	0.10	5380
Training	0.03	12652	0.03	5380
Other nonworking	0.05	12652	0.05	5380
2017				
Age	58.64	8064	57.68	3422
College	0.25	8042	0.28	3420
Vocational training	0.63	8060	0.66	3422
Secondary schooling	0.08	8050	0.04	3412
Intermediate schooling	0.03	8064	0.01	3422
Male	0.46	8064	0.46	3422
Number of children	0.32	8064	0.34	3422
Number of adults	2.79	8064	2.70	3422
Married	0.64	8063	0.60	3422
Divorced	0.10	8063	0.11	3422
Married but separated	0.03	8063	0.02	3422
Widowed	0.09	8063	0.10	3422
Log of net HH monthly income, in 2011 Euro	7.95	7673	7.77	3332
Civil servant	0.05	8063	0.03	3422
Self-employed	0.06	8064	0.05	3422
White-collar worker	0.34	8064	0.32	3418
Unemployed	0.02	8064	0.04	3422
Retired	0.33	8064	0.31	3422
Maternity	0.01	8064	0.01	3422
Nonworking	0.07	8064	0.07	3422
Training	0.00	8064	0.00	3422
Other nonworking	0.02	8064	0.02	3422

Table A4: Mean of the independent variables, by East/West and Year, balanced sample

	West		East	
	Mean	N	Mean	N
1997				
Age	42.52	1390	42.48	937
College	0.16	1390	0.32	937
Vocational training	0.67	1390	0.59	937
Secondary schooling	0.12	1390	0.06	937
Intermediate schooling	0.04	1390	0.02	937
Male	0.48	1390	0.46	937
Number of children	0.66	1390	0.61	937
Number of adults	3.81	1390	3.13	937
Married	0.67	1390	0.68	937
Divorced	0.06	1390	0.07	937
Married but separated	0.02	1390	0.02	937
Widowed	0.03	1390	0.02	937
Log of net HH monthly income, in 2011 Euro	8.01	1390	7.80	937
Civil servant	0.09	1390	0.02	937
Self-employed	0.06	1390	0.05	937
White-collar worker	0.40	1390	0.37	937
Unemployed	0.04	1390	0.12	937
Retired	0.03	1390	0.02	937
Maternity	0.02	1390	0.02	937
Nonworking	0.14	1390	0.08	937
Training	0.02	1390	0.02	937
Other nonworking	0.04	1390	0.05	937
2002				
Age	47.52	1390	47.48	937
College	0.19	1390	0.33	937
Vocational training	0.70	1390	0.63	937
Secondary schooling	0.09	1390	0.04	937
Intermediate schooling	0.02	1390	0.00	937
Male	0.48	1390	0.46	937
Number of children	0.59	1390	0.45	937
Number of adults	3.71	1390	3.31	937
Married	0.71	1390	0.70	937
Divorced	0.08	1390	0.08	937
Married but separated	0.02	1390	0.02	937
Widowed	0.04	1390	0.04	937
Log of net HH monthly income, in 2011 Euro	8.04	1390	7.84	937
Civil servant	0.07	1390	0.02	937
Self-employed	0.06	1390	0.05	937
White-collar worker	0.41	1390	0.35	937
Unemployed	0.03	1390	0.09	937
Retired	0.08	1390	0.07	937
Maternity	0.02	1390	0.02	937
Nonworking	0.14	1390	0.12	937
Training	0.01	1390	0.01	937
Other nonworking	0.04	1390	0.04	937
2017				
Age	62.52	1390	62.48	937
College	0.21	1390	0.35	937
Vocational training	0.70	1390	0.62	937
Secondary schooling	0.08	1390	0.02	937
Intermediate schooling	0.02	1390	0.00	937
Male	0.48	1390	0.46	937
Number of children	0.21	1390	0.21	937
Number of adults	3.53	1390	3.05	937
Married	0.70	1390	0.68	937
Divorced	0.10	1390	0.09	937
Married but separated	0.02	1390	0.02	937
Widowed	0.10	1390	0.12	937
Log of net HH monthly income, in 2011 Euro	7.99	1390	7.79	937
Civil servant	0.05	1390	0.02	937
Self-employed	0.06	1390	0.06	937
White-collar worker	0.32	1390	0.28	937
Unemployed	0.02	1390	0.02	937
Retired	0.37	1390	0.39	937
Maternity	0.00	1390	0.00	937
Nonworking	0.08	1390	0.08	937
Training	0.00	1390	0.00	937
Other nonworking	0.02	1390	0.02	937

T-statistics

Table A5: χ^2 of the Wald test, which tests the equality of the parameters estimated in Table 1. Stars indicate that the parameters are significantly different at the 1% (***), 5% (**) or 10% (*) level.

Responsibility for financial security...	Year 2002 - Year 2017	East*2002 - East*2017
...when unemployed	62.9***	30.5***
...when sick	34.7***	1.24
...of the family	45.0***	0.42
...when old	81.5***	0.5
...when requiring care	64.9***	0.0

Baseline regression without controls

Table A6: Baseline regressions omitting control variables

	Dependent variable: Responsibility for financial security...				
	...when unemployed	...when sick	...of the family	...when old	...when requiring care
East	0.470***	0.464***	0.428***	0.450***	0.406***
	(0.028)	(0.026)	(0.027)	(0.026)	(0.026)
Year 2002	0.021	0.113***	-0.031	-0.054***	0.087***
	(0.019)	(0.019)	(0.019)	(0.019)	(0.019)
East*2002	-0.129***	-0.131***	-0.075**	-0.132***	-0.151***
	(0.034)	(0.032)	(0.032)	(0.031)	(0.032)
Year 2017	0.156***	0.190***	0.072***	0.073***	0.197***
	(0.022)	(0.021)	(0.022)	(0.021)	(0.021)
East*2017	-0.322***	-0.161***	-0.095***	-0.146***	-0.150***
	(0.039)	(0.036)	(0.036)	(0.036)	(0.037)
Observations	38779	38820	38743	38858	38856
Individuals	26108	26133	26092	26144	26140
Log likelihood	-23816	-26186	-25233	-26152	-26629

Probit regressions. The dependent variable is an indicator variable that takes the value one if the household responds *only the state* or *mostly the state* to the question of who should be responsible for the financial security of different groups.
* $p < 0.10$, ** $p < 0.05$, *** $p < 0.01$. Robust clustered standard errors in parentheses.

Marginal effects

Table A7: Percentage point difference in probability of being pro-state in 2002 and 2017 compared to 1997. Based on the coefficients of East, East*2002 and East*2017 from Table 1.

Responsibility for financial security...	1997	2002	2017
...when unemployed	15.1	-4.7	-11.5
...when sick	17.8	-6.5	-8.0
...of the family	15.5	-3.6	-4.4
...when old	16.6	-6.0	-7.0
...when requiring care	14.8	-6.3	-6.3

Table A8: Percentage point difference in probability of being pro-state in 2002 and 2017 compared to 1997. Based on the coefficients of East, East*2002 and East*2017 from Table 5.

Responsibility for financial security...	1997	2002	2017
...when unemployed	13.8	-4.8	-5.3
...when sick	15.3	-5.3	-6.3
...of the family	15.0	-0.9	-1.2
...when old	10.6	-1.0	1.4
...when requiring care	11.7	-6.1	-6.3

Testing for Selective Attrition

Table A9: Attrition

	In sample in 1997 and 2002	In sample in 2002 and 2017
...when unemployed	0.060*	0.025
	(0.036)	(0.030)
...when sick	-0.037	-0.016
	(0.036)	(0.030)
...of the family	0.011	0.005
	(0.034)	(0.029)
...when old	-0.074**	-0.044
	(0.037)	(0.032)
...when requiring care	-0.030	0.057**
	(0.034)	(0.029)
East	-0.017	0.320***
	(0.033)	(0.028)
Age	-0.064***	0.120***
	(0.023)	(0.023)
Age squared	0.002***	-0.001**
	(0.000)	(0.000)
Age cubed	-0.000***	-0.000
	(0.000)	(0.000)
College	0.014	1.308***
	(0.121)	(0.227)
Vocational training	-0.002	1.202***
	(0.114)	(0.225)
Secondary schooling	-0.036	1.139***
	(0.116)	(0.227)
Intermediate schooling	-0.048	0.863***
	(0.129)	(0.241)
Male	-0.068**	-0.069***
	(0.032)	(0.027)
Number of children	0.035*	0.013
	(0.021)	(0.018)
Number of adults	0.027***	0.155***
	(0.009)	(0.008)
Married	0.114**	-0.081*
	(0.051)	(0.046)
Divorced	-0.022	-0.090
	(0.074)	(0.062)
Married but separated	0.155	-0.098
	(0.114)	(0.093)
Widowed	0.084	0.002
	(0.080)	(0.076)
Log HH income	-0.031	-0.012
	(0.036)	(0.029)
Civil servant	-0.050	0.174**
	(0.081)	(0.068)
Self-employed	-0.111	0.001
	(0.072)	(0.061)
White-collar worker	0.106**	0.134***
	(0.047)	(0.039)
Unemployed	0.137*	-0.097
	(0.072)	(0.063)
Retired	-0.171*	0.005
	(0.088)	(0.077)
Maternity	-0.018	0.295***
	(0.118)	(0.097)
Nonworking	-0.031	-0.014
	(0.062)	(0.050)
Training	0.045	0.256**
	(0.105)	(0.112)
Other nonworking	-0.074	0.145**
	(0.074)	(0.067)
Observations	9189	16435
Log likelihood	-4861	-6434

Probit regressions. The dependent variable is an indicator variable that takes the value one if the household is present in both sample years. Omitted categories are fewer than nine years of schooling, female, single, blue-collar worker, and employed.

* $p < 0.10$, ** $p < 0.05$, *** $p < 0.01$. Robust standard errors clustered on individual level in parentheses.

Age effects

Table A10: Regressions with age interacted with East

	Dependent variable: Responsibility for financial security...				
	...when unemployed	...when sick	...of the family	...when old	...when requiring care
East	0.050	0.076	0.036	-0.105**	0.026
	(0.053)	(0.049)	(0.049)	(0.049)	(0.048)
Year 2002	0.051**	0.167***	0.003	-0.014	0.119***
	(0.020)	(0.020)	(0.021)	(0.020)	(0.020)
East*2002	-0.161***	-0.189***	-0.117***	-0.187***	-0.179***
	(0.035)	(0.033)	(0.033)	(0.033)	(0.033)
Year 2017	0.235***	0.309***	0.165***	0.201***	0.298***
	(0.024)	(0.024)	(0.024)	(0.024)	(0.024)
East*2017	-0.452***	-0.315***	-0.228***	-0.338***	-0.261***
	(0.042)	(0.040)	(0.040)	(0.040)	(0.040)
Age	-0.001	-0.001	-0.003***	-0.003***	-0.005***
	(0.001)	(0.001)	(0.001)	(0.001)	(0.001)
East*age	0.009***	0.009***	0.009***	0.012***	0.008***
	(0.001)	(0.001)	(0.001)	(0.001)	(0.001)
All control variables	Yes	Yes	Yes	Yes	Yes
Observations	36564	36599	36532	36636	36637
Individuals	24819	24840	24801	24847	24848
Log likelihood	-22141	-24375	-23591	-24234	-24921

Probit regressions. The dependent variable is an indicator variable that takes the value one if the household responds *only the state* or *mostly the state* to the question of who should be responsible for the financial security of different groups. Included as controls are number of children and number of adults in household, logarithm of household income, and dummies for education, sex, marital status, employment status, and occupation.
* $p < 0.10$, ** $p < 0.05$, *** $p < 0.01$. Robust clustered standard errors in parentheses.

Parents' preferences

Table A11: Regressions with those who were born in 1990-1999, information about at least one parent's preferences is present

	Dependent variable: Responsibility for financial security...				
	...when unemployed	...when sick	...of the family	...when old	...when requiring care
Born in East States	-0.207	0.008	-0.167	-0.044	-0.050
	(0.205)	(0.194)	(0.189)	(0.193)	(0.190)
Born to Eastern parents	0.227	0.120	0.426**	0.421**	0.176
	(0.204)	(0.191)	(0.187)	(0.190)	(0.187)
Age	0.008	0.002	0.013	0.024	0.044***
	(0.016)	(0.016)	(0.016)	(0.016)	(0.016)
Male	-0.060	-0.008	0.078	-0.095	-0.045
	(0.092)	(0.088)	(0.088)	(0.088)	(0.088)
Unemployed	0.439	0.110	-0.056	-0.021	-0.073
	(0.299)	(0.264)	(0.255)	(0.266)	(0.263)
Maternity	0.585	0.957	-0.504	-0.089	0.772
	(0.590)	(0.590)	(0.528)	(0.490)	(0.605)
Training	0.191*	0.092	-0.040	0.032	0.122
	(0.111)	(0.106)	(0.106)	(0.106)	(0.107)
Other nonworking	0.457***	0.274*	0.104	0.154	-0.003
	(0.166)	(0.154)	(0.155)	(0.152)	(0.152)
Observations	846	843	844	845	846
Log likelihood	-518	-575	-573	-575	-573

Probit regressions. The dependent variable is an indicator variable that takes the value one if the household responds *only the state* or *mostly the state* to the question of who should be responsible for the financial security of different groups.
* $p < 0.10$, ** $p < 0.05$, *** $p < 0.01$. Robust standard errors in parentheses.

Table A12: Regressions with those who were born in 1990-1999, including mother's preferences

	Dependent variable: Responsibility for financial security...				
	...when unemployed	...when sick	...of the family	...when old	...when requiring care
Born in East States	-0.207	0.077	-0.145	-0.076	0.008
	(0.210)	(0.208)	(0.198)	(0.207)	(0.201)
Born to Eastern parents	0.206	0.012	0.367*	0.422**	0.095
	(0.207)	(0.204)	(0.195)	(0.203)	(0.197)
Mother pro-state in 2017	0.494***	0.275***	0.408***	0.471***	0.218**
	(0.101)	(0.091)	(0.091)	(0.091)	(0.090)
Age	-0.001	0.007	0.008	0.009	0.040**
	(0.017)	(0.016)	(0.016)	(0.017)	(0.016)
Male	-0.017	-0.001	0.062	-0.117	-0.023
	(0.095)	(0.091)	(0.091)	(0.091)	(0.090)
Unemployed	0.408	0.161	0.022	-0.130	-0.029
	(0.306)	(0.274)	(0.269)	(0.274)	(0.274)
Maternity	0.540	0.835	-0.415	0.285	0.707
	(0.645)	(0.594)	(0.522)	(0.558)	(0.644)
Training	0.155	0.045	-0.063	0.015	0.128
	(0.114)	(0.109)	(0.110)	(0.110)	(0.110)
Other nonworking	0.504***	0.338**	0.159	0.140	0.061
	(0.181)	(0.164)	(0.163)	(0.162)	(0.160)
Observations	801	796	798	799	798
Log likelihood	-479	-537	-531	-531	-539

Probit regressions. The dependent variable is an indicator variable that takes the value one if the household responds *only the state* or *mostly the state* to the question of who should be responsible for the financial security of different groups.
* $p < 0.10$, ** $p < 0.05$, *** $p < 0.01$. Robust standard errors in parentheses.

Table A13: Regressions with those who were born in 1990-1999, including father's preferences

	Dependent variable: Responsibility for financial security...				
	...when unemployed	...when sick	...of the family	...when old	...when requiring care
Born in East States	-0.303	-0.175	-0.329	0.019	-0.132
	(0.229)	(0.224)	(0.218)	(0.219)	(0.220)
Born to Eastern parents	0.184	0.218	0.524**	0.399*	0.195
	(0.226)	(0.220)	(0.215)	(0.213)	(0.213)
Father pro-state in 2017	0.342***	0.313***	0.422***	0.239**	0.390***
	(0.110)	(0.101)	(0.104)	(0.102)	(0.100)
Age	0.011	0.013	0.028	0.028	0.055***
	(0.018)	(0.018)	(0.018)	(0.018)	(0.018)
Male	0.015	0.032	0.130	-0.076	-0.054
	(0.104)	(0.100)	(0.101)	(0.100)	(0.101)
Unemployed	0.577	0.354	-0.006	-0.067	0.085
	(0.373)	(0.317)	(0.297)	(0.314)	(0.299)
Maternity	0.000	0.000	-0.738	-0.460	0.566
	(.)	(.)	(0.750)	(0.594)	(0.724)
Training	0.238*	0.102	-0.057	0.098	0.105
	(0.127)	(0.121)	(0.121)	(0.121)	(0.122)
Other nonworking	0.408**	0.337*	-0.021	0.086	0.041
	(0.181)	(0.174)	(0.177)	(0.171)	(0.173)
Observations	649	644	654	652	654
Log likelihood	-397	-437	-432	-440	-436

Probit regressions. The dependent variable is an indicator variable that takes the value one if the household responds *only the state* or *mostly the state* to the question of who should be responsible for the financial security of different groups.
* $p < 0.10$, ** $p < 0.05$, *** $p < 0.01$. Robust standard errors in parentheses.

OLS results

Table A14: Balanced sample: regressions with individuals who answer in 1997, 2002 and 2017

	Dependent variable: Responsibility for financial security...				
	...when unemployed	...when sick	...of the family	...when old	...when requiring care
East	0.138***	0.152***	0.152***	0.107***	0.117***
	(0.020)	(0.022)	(0.021)	(0.022)	(0.022)
Year 2002	0.026	0.072***	0.002	-0.004	0.074***
	(0.017)	(0.017)	(0.017)	(0.017)	(0.018)
East*2002	-0.048**	-0.050*	-0.010	-0.011	-0.060**
	(0.024)	(0.026)	(0.026)	(0.026)	(0.027)
Year 2017	0.054***	0.119***	0.033	0.059***	0.132***
	(0.020)	(0.020)	(0.021)	(0.021)	(0.021)
East*2017	-0.057**	-0.059**	-0.008	0.017	-0.063**
	(0.025)	(0.028)	(0.027)	(0.028)	(0.029)
All control variables	Yes	Yes	Yes	Yes	Yes
Observations	6981	6981	6981	6981	6981
Individuals	2327	2327	2327	2327	2327
Log likelihood	-4311	-4880	-4758	-4854	-4972

Linear probability model. The dependent variable is an indicator variable that takes the value one if the household responds *only the state* or *mostly the state* to the question of who should be responsible for the financial security of different groups. Included as controls are cubic function in age, number of children and number of adults in household, logarithm of household income, and dummies for education, sex, marital status, employment status, and occupation. * $p < 0.10$, ** $p < 0.05$, *** $p < 0.01$. Robust clustered standard errors in parentheses.

Table A15: Basic regressions

	Dependent variable: Responsibility for financial security...				
	...when unemployed	...when sick	...of the family	...when old	...when requiring care
East	0.146***	0.179***	0.161***	0.170***	0.149***
	(0.010)	(0.011)	(0.011)	(0.011)	(0.011)
Year 2002	0.016**	0.060***	-0.001	-0.008	0.044***
	(0.007)	(0.007)	(0.007)	(0.008)	(0.008)
East*2002	-0.043***	-0.064***	-0.040***	-0.063***	-0.063***
	(0.011)	(0.013)	(0.012)	(0.012)	(0.013)
Year 2017	0.073***	0.103***	0.047***	0.058***	0.105***
	(0.009)	(0.009)	(0.009)	(0.009)	(0.009)
East*2017	-0.111***	-0.077***	-0.044***	-0.070***	-0.064***
	(0.013)	(0.015)	(0.014)	(0.015)	(0.015)
Age	-0.007*	0.009**	-0.003	0.003	0.004
	(0.004)	(0.004)	(0.004)	(0.004)	(0.004)
Age squared(*10^3)	0.172**	-0.144*	0.079	-0.058	-0.083
	(0.075)	(0.079)	(0.078)	(0.079)	(0.080)
Age cubed(*10^5)	-0.117***	0.073	-0.058	0.030	0.047
	(0.045)	(0.048)	(0.047)	(0.048)	(0.049)
College	-0.066***	-0.114***	-0.084***	-0.121***	-0.049**
	(0.021)	(0.023)	(0.023)	(0.023)	(0.023)
Vocational training	-0.053***	-0.086***	-0.094***	-0.088***	-0.041*
	(0.020)	(0.022)	(0.022)	(0.022)	(0.022)
Secondary schooling	-0.053***	-0.057**	-0.058**	-0.051**	-0.020
	(0.020)	(0.022)	(0.022)	(0.023)	(0.023)
Intermediate schooling	-0.062***	-0.073***	-0.101***	-0.069***	-0.046*
	(0.023)	(0.025)	(0.025)	(0.025)	(0.025)
Male	-0.013**	-0.017***	-0.006	0.001	0.011*
	(0.005)	(0.006)	(0.006)	(0.006)	(0.006)
Number of children	0.016***	0.015***	0.027***	0.011***	0.007*
	(0.004)	(0.004)	(0.004)	(0.004)	(0.004)
Number of adults	0.008***	0.011***	0.008***	0.008***	0.004**
	(0.002)	(0.002)	(0.002)	(0.002)	(0.002)
Married	0.010	0.019*	0.009	0.016*	0.017*
	(0.009)	(0.010)	(0.009)	(0.010)	(0.010)
Divorced	0.002	-0.012	0.009	0.000	0.001
	(0.012)	(0.013)	(0.013)	(0.013)	(0.013)
Married but separated	-0.008	-0.003	0.001	0.008	0.019
	(0.018)	(0.019)	(0.019)	(0.020)	(0.020)
Widowed	0.005	0.005	0.004	0.007	0.013
	(0.014)	(0.014)	(0.014)	(0.014)	(0.014)
Log HH income	-0.053***	-0.079***	-0.062***	-0.090***	-0.066***
	(0.006)	(0.006)	(0.006)	(0.006)	(0.006)
Civil servant	-0.070***	-0.082***	0.018	-0.045***	-0.066***
	(0.015)	(0.015)	(0.015)	(0.015)	(0.015)
Self-employed	-0.119***	-0.121***	-0.093***	-0.143***	-0.094***
	(0.013)	(0.013)	(0.013)	(0.013)	(0.013)
White-collar worker	-0.015*	-0.019**	-0.009	-0.039***	-0.030***
	(0.008)	(0.009)	(0.008)	(0.009)	(0.009)
Unemployed	0.033***	0.002	0.047***	0.002	-0.006
	(0.012)	(0.014)	(0.014)	(0.014)	(0.014)
Retired	-0.042***	-0.044***	0.002	-0.027*	-0.013
	(0.013)	(0.014)	(0.014)	(0.014)	(0.014)
Maternity	-0.000	-0.027	0.007	-0.058**	-0.027
	(0.020)	(0.023)	(0.022)	(0.022)	(0.023)
Nonworking	-0.013	-0.014	0.023**	-0.012	-0.008
	(0.010)	(0.011)	(0.011)	(0.011)	(0.011)
Training	-0.033	-0.026	-0.075***	-0.028	-0.026
	(0.021)	(0.022)	(0.021)	(0.022)	(0.022)
Other nonworking	-0.004	-0.029**	-0.001	-0.020	-0.038**
	(0.013)	(0.015)	(0.014)	(0.015)	(0.015)
Observations	36564	36599	36532	36636	36637
Individuals	24819	24840	24801	24847	24848
Log likelihood	-23233	-25615	-24801	-25524	-26157

Linear probability model. The dependent variable is an indicator variable that takes the value one if the household responds *only the state* or *mostly the state* to the question of who should be responsible for the financial security of different groups. Omitted categories are fewer than nine years of schooling, female, single, blue-collar worker, and employed. * $p < 0.10$, ** $p < 0.05$, *** $p < 0.01$. Robust clustered standard errors in parentheses.
